# Activation-tagging in *indica* rice identifies a novel transcription factor subunit, *NF-YC13* associated with salt tolerance

**DOI:** 10.1038/s41598-017-10022-9

**Published:** 2017-08-24

**Authors:** P. Manimaran, S. Venkata Reddy, Mazahar Moin, M. Raghurami Reddy, Poli Yugandhar, S. S. Mohanraj, S. M. Balachandran, P. B. Kirti

**Affiliations:** 1 Department of Plant Sciences, University of Hyderabad, Hyderabad, 5000046 India; 2 Indian Institute of Rice Research, Rajendranagar, Hyderabad, 500030 India

## Abstract

Nuclear factor Y (NF-Y) is a heterotrimeric transcription factor with three distinct NF-YA, NF-YB and NF-YC subunits. It plays important roles in plant growth, development and stress responses. We have reported earlier on development of gain-of-function mutants in an *indica* rice cultivar, BPT-5204. Now, we screened 927 seeds from 70 *Ac/Ds* plants for salinity tolerance and identified one activation-tagged salt tolerant DS plant (DS-16, T_3_ generation) that showed enhanced expression of a novel ‘histone-like transcription factor’ belonging to rice NF-Y subfamily C and was named as *OsNF-YC13*. Localization studies using GFP-fusion showed that the protein is localized to nucleus and cytoplasm. Real time expression analysis confirmed upregulation of transcript levels of *OsNF-YC13* during salt treatment in a tissue specific manner. Biochemical and physiological characterization of the DS-16 revealed enhanced K^+^/Na^+^ ratio, proline content, chlorophyll content, enzymes with antioxidant activity etc. DS-16 also showed transcriptional up-regulation of genes that are involved in salinity tolerance. *In-silico* analysis of *OsNF-YC13* promoter region evidenced the presence of various key stress-responsive *cis*-regulatory elements. OsNF-YC13 subunit alone does not appear to have the capacity for direct transcription activation, but appears to interact with the B- subunits in the process of transactivation.

## Introduction

Rice (*Oryza sativa* L.) is a major staple food crop consumed globally by more than three billion people. Various abiotic stresses such as extreme temperature, water deficiency, high salinity and submergence adversely affect rice crop growth and productivity. Soil salinity, in addition to other abiotic stresses, is one of the most severe problems in agriculture and partly responsible for lower rice productivity^1^. Increasing soil salinization in irrigated areas has necessitated the identification of genes that confer resistance to salinity either by conventional breeding or genetic engineering^2^.

Salinity is a complex trait, which affects almost every aspect of the physiology and biochemistry of plants both at whole plant and cellular levels through osmotic and ionic stresses^3, 4^. Absorption of excessive salt inhibits both root and shoot growth, reduces reproductive activity and affects viability of plants. To counter salinity stress, plant cells have several defense mechanisms. These include secretion of toxic levels of sodium ions from the cytosol to the apoplast or into the vacuole mediated by the Na^+^/H^+^ exchanger, SOS1 located at the plasma membrane and the vacuolar Na^+^/H^+^ antiporter NHX1, respectively^5^.

Recent molecular genetic studies of rice have also revealed several factors involved in mechanisms that ensure plant growth under saline conditions particularly root using loss-of-function mutants^6, 7^. Development of salinity tolerant rice varieties through transgenic approach is an efficient option to overcome salinity stress. Several studies reported that overexpression of stress-induced/transcription factor/transporter genes from rice or other plant species conferred salinity tolerance in rice. Some of these genes conferring enhanced salt tolerance in rice are *OsLEA4* ^8^, *OsPP1a* ^9^, *OsGly1* ^10^, *OsJRL* ^11^. Although transgenic plants targeting the modification of a single gene have shown improved tolerance to salinity, progress in this area of research is hampered due to lack of complete understanding of the molecular basis of salt tolerance, which involves a complex network of genes operating in close coordination^12, 13^. Hence, there is still the possibility of identifying further novel genes through functional genomic approaches, which facilitate the identification of novel genes and their functions that would greatly strengthen our efforts in the development of stress tolerance (both biotic/abiotic) in rice. In order to identify novel candidate gene(s), an efficient gain-of-function mutagenesis strategy called ‘activation-tagging’ (AT) was employed in an *indica* rice variety. AT is a powerful tool used for generating huge numbers of independent transformed lines with the possibility of gain-of-function mutagenesis and identification of novel genes. High throughput profiling of these activation-tagging lines provides useful resources to identify genes that are involved in regulatory or biosynthesis pathways^14^. This system uses tetramer copies of cauliflower mosaic virus (CaMV) 35 S enhancers^15^ and the enhancers upon integration in the recipient plant genome can function in either orientation at the place of insertion, thereby causing transcriptional activation of nearby genes resulting in dominant gain-of-function mutations^16^. There are several reports citing this technique with the development of AT lines in *Arabidopsis* ^17, 18^, *japonica* rice^16, 19^, *indica* rice^20^, tomato^21, 22^, poplar^23^, strawberry, potato^24^, barley^25^ and sorghum^26^.

Transcription factors (TFs) are a group of regulatory proteins that regulate the expression of genes under different environmental stress conditions^27, 28^. These TFs bind to specific *cis*-elements in the promoter regions of stress-responsive genes and initiate complex signaling cascades in various biosysnthetic pathways including those in response to abiotic stresses^29^. Nuclear factor Y (NF-Y), also known as Heme activator protein (HAP) or CCAAT binding factor (CBF) is a heterotrimeric complex transcription factor with high affinity and sequence specificity to a *cis*-element, CCAAT box in eukaryote promoters and has been reported to play roles in the activation of diverse genes^30^. This heterotrimeric complex is composed of three subunits: NF-YA, NF-YB and NF-YC. A unique pattern of heterotrimer formation of NF-Y complex happens first in cytoplasm with NF-YB and NF-YC forming a heterodimer, which subsequently translocates to the nucleus, where the dimer interacts with NF-YA subunit to form a mature NF-Y transcription factor^31, 32^. It is binding to a specific sequence (CCAAT) in a promoter results in either positive or negative transcriptional regulation of genes under its control^33^.

In most eukaryotic genomes, the NF-Y is encoded by a single gene, whereas in plant genomes, each subunit is encoded by a family of genes^34^. Genome wide survey of NF-Ys in *Arabidopsis* revealed 10 NF-YA, 13 NF-YB, and 13 NF-YC genes^35–37^. In wheat, 37 NF-Y and Dr1 genes (10 NF-YA, 11 NF-YB, 14 NF-YC and 2 Dr1) were identified^38^. Recently, several groups have identified NF-Y genes in different plant species such as 68 NF-Ys in soybean^39^, 59 NF-Ys in tomato^40^, 27 in *Prunus mume* ^41^, 34 in *Vitis vinifera* ^42^. In rice, 28 NF-Y genes were identified including 10 NF-YAs, 11 NF-YBs, 7 NF-YCs^43^. However, Yang and co-workers^44^ reported the identification of some other genes in the Rice Genome Annotation Project (RGAP) database, which were included in the NF-Y family enlarging the total to 40 putative NF-Y genes (11 NF-YA, 13 NF-YB, and 16 NF-YC). Reports suggest that NF-Y gene encoded proteins undertake combinatorial and functional diversity like plant growth, development and stress responses. However, the exact molecular mechanism of NF-Y towards plant development and stress response is unclear, which needs to be explored^45, 46^.

The objective of the current investigation is to identify salt-tolerant rice lines among the activation-tagged *indica* rice (variety BPT 5204, Samba Mahsuri) lines and characterize the flanking sequences on either side of the integration of *Ds* element carrying multiple enhancers and study the expression of genes in order to identify the tagged genes. In this communication, we report on the identification and characterization of a novel nuclear factor Y gene (*NF-YC13*) in one of the identified salt tolerant AT lines that has been shown to impart salt tolerance in rice.

## Results

### Screening and confirmation of identified salt-tolerant *DS* lines

**Figure 1:**
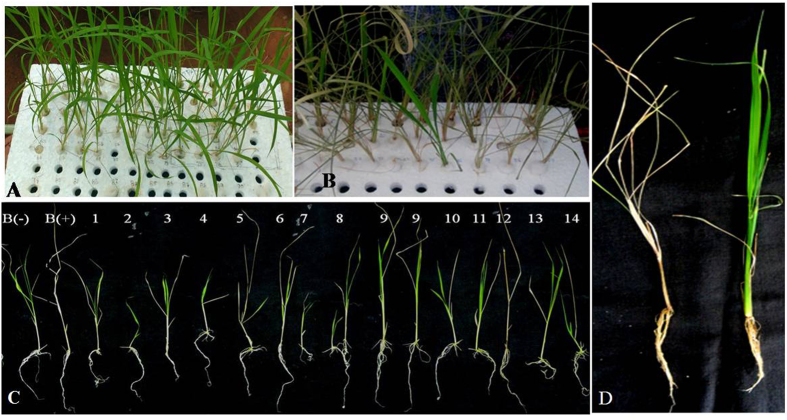
Hydroponic screening of *Ds* plants for salinity tolerance. (**A**) Two weeks old seedlings were in Yoshida’s solution before stress treatment. (**B**) Seedlings in Yoshida’s solution supplemented with 150 mM NaCl. (**C**) Salt tolerant *Ds* plants after NaCl treatment. (**D**) Plants after recovery (Represented line). B(−): WT-BPT without stress; B( + ): WT-BPT with NaCl stress treatment; 1–14: *Ds* lines showing tolerance after two weeks of NaCl stress.

The rice variety BPT 5204 was transformed with pSQ5 vector, which carries the *Ac* element with the *hph* gene for plant selection, *Ds* element carrying multiple 35S enhancers and *RFP*gene^47^ and the transgenic plants carrying both *Ac*/*Ds* elements and stable *Ds* lines were developed^20^. A total of 1828 seeds from 70 transgenic *Ac/Ds* element carrying plants in T_3_ generation were inoculated in ½ MS medium. About 927 seeds germinated into seedlings that were subsequently transferred to Yoshida’s culture solution for 3–5 d in the transgenic greenhouse (Fig. 1A). At two or three-leaf stage (15–20 d), seedlings were treated with 150 mM sodium chloride at pH 5.8 for two weeks. In the salt treatment, fresh solution was replaced every three days to maintain the pH 5.8. Along with the transgenic plants, seedlings of wild type BPT-5204 were also transferred and treated in the same tray. On the third day of salt stress, the older leaves started rolling and drying up. During the stress treatment, some of the stable *Ds* plants showed tolerance where the leaves remained green without the burning symptoms, whereas the susceptible transgenic plants along with the controls were completely dried and died (Fig. 1B and C). After stress treatment (15 d), the tolerant plants were transferred to fresh Yoshida’s culture solution and allowed for 1–2 weeks for recovery from the salt stress treatment (Fig. 1D). Out of 927 seedlings (70 lines), 59 showed salt tolerance to varying levels and recovered. After complete recovery of the 59 plants, 35 plants showed normal growth in vegetative as well as reproductive stages, while the remaining 24 plants showed retarded growth.

The 35 salt tolerant transgenic plants were screened for the presence of *Ac*/*Ds* elements through PCR. In this procedure, the amplification with *hph* (Hygromycin phospho transferase) gene specific primers indicates the presence of the *Ac* element. Similarly, amplification with *RFP* (Red fluorescent protein) gene specific primers indicates the presence of *Ds* element in the corresponding plant. From PCR analysis 30 out of 35 plants were found to be stable *Ds* element carrying plants whereas the remaining 5 plants represented *Ac*-*Ds* plants (supplementary Fig. 1A).

### Identification of insertion site and flanking sequences on the chromosome

**Figure 2:**
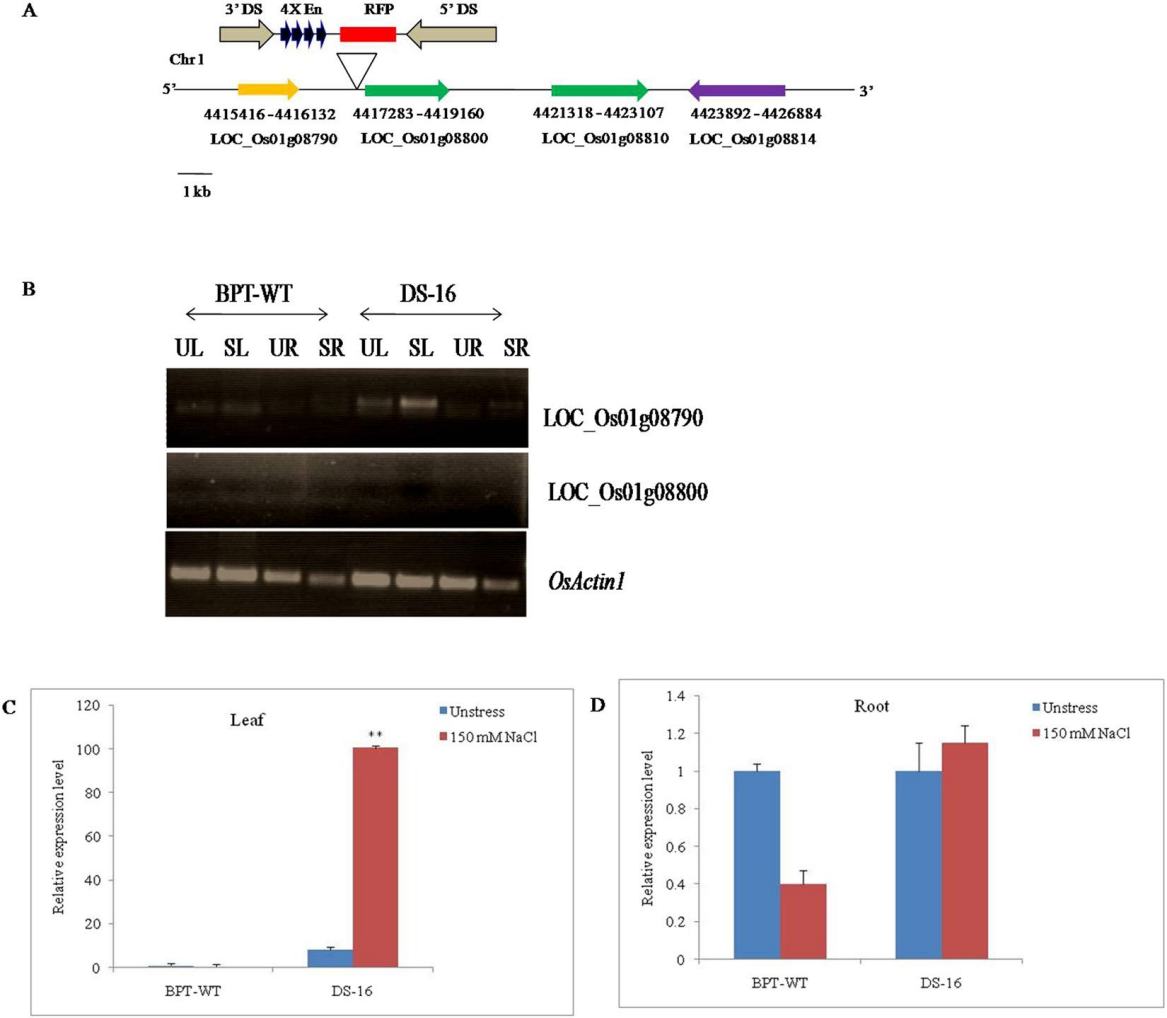
Physical mapping of insertion site of *Ds*-Enhancer (DS-En) elements and expression analysis of *NF-YC13* in salt tolerant activation-tagged DS-16 line. (**A**) *Ds* element was inserted in the intergenic region on chromosome 1 and location of neighboring four putative genes (up to 20 kb region) near the insertion site. (**B**) Semi-quantitative RT-PCR analysis of activation-tagged DS-16 line. Two weeks old seedlings grown in Yoshida’s solution supplemented with 150 mM NaCl for 15 d and those without NaCl supplementation used as control. One of the genes, LOC_Os01g08790 shows expression during control and NaCl stress conditions while no expression in leaf and root of WT-BPT and LOC_Os01g08800 respectively. (**C**,**D**) Quantitative real time-PCR analysis of expression of *NF-YC13* in the activation-tagged DS-16 line in leaf and root tissues. *OsActin1* was used as a reference gene for quantitative RT-PCR. Means of three independent samples and standard errors are presented. ** and * indicates significant difference at P < 0.01 and P < 0.05.

Twenty best salt tolerant plants were selected out of the 30 selected *Ds* lines and genomic DNA was isolated followed by a three-step TAIL PCR to identify the flanking sequences. Twenty transgenic plants gave specific TAIL-PCR products above 500 to 750 bp (supplementary Fig. 1B), which were subsequently gel purified, cloned and sequenced. The sequences were analyzed through the rice annotation sites at Michigan State University (http://rice.plantbiology.msu.edu), The Institute of Genomics Research (http://blast.jcvi.org/euk-blast/) and RAP-DB (http://rapdb.dna.affrc.go.jp/). All the transgenic plants showed independent *Ds* insertion events with the *Ds* element getting inserted at different loci on the rice chromosomes (Supplementary Table 2). Apart from mapping the insertion of *Ds* element on the corresponding rice chromosome, a 10 kb region on either side of the insertion site was also mapped, which aided in the identification of the genes flanking the *Ds* element insertions (Supplementary Fig. 2). The plant DS-16 showed the insertion on chromosome 1, and the BLAST results showed that the insertion on chromosome 1 was at an intergenic region near LOC_Os01g08800 encoding a putative cytochrome P450 gene (Fig. 2A). A 10 kb region on either side of the *Ds* insertion, carried the genes histone-like transcription factor and archeal histone (LOC_Os01g08790), cytochrome P450 (LOC_Os01g08810), an expressed protein (LOC_Os01g08814). Similarly, the insertion sites of other different salt-tolerant DS lines and details of nearby genes located are shown (Supplementary Table 2). Further, the left boarder (LB) flanking sequence analysis was performed for both PDS-16 plant (Parent of DS-16, with both *Ac/Ds* elements) and *Ac* plant (after transposition of *Ds* element from T-DNA) to confirm the stability of the *Ac* element. Results revealed that the T-DNA was located at a non-coding region on chromosome number 12 (position at approximately 24871372). Nearby gene was identified as expressed protein (LOC_Os12g40180) with unknown function towards downstream of integration loci. There was no positional variation in T-DNA, before and after the transposition.

### Enhanced expression of tagged gene in DS-16 and analysis of other stress-responsive genes

Out of all salt tolerant *Ds* plants, the plant DS-16 was selected based on its salt tolerance and the insertion of the enhancer carrying *Ds* element in the intergenic region without disturbing any genes, whereas the insertion site was in the intergenic region in other *Ds* plants. Also the tagged gene at the locus, LOC_Os01g08790 was yet to be reported/characterized for abiotic stress. Hence, the DS-16 plant was analyzed for the expression profile of the neighboring genes identified near the insertion site of the *Ds* element for the possible identification of the activated gene(s), if any. Analysis of the RNA samples of both plants, control and NaCl stressed conditions of DS-16 and WT-BPT respectively was carried out using semi-quantitative and qRT-PCR. In DS-16, four putative genes, LOC_Os01g08790, LOC_Os01g08800, LOC_Os01g08810 and LOC_Os01g08814 were located within a 20 kb region near the *Ds* insertion site on either side (Fig. 2A). The *Ds* element was identified at a site 636 bp (4416647–4417283) upstream of the of the LOC_Os01g08800 gene, which encodes a putative Cytochrome P450 on chromosome 1 (Fig. 2A). At the same time, LOC_Os01g08790 gene (putative, histone-like transcription factor) was located 1.4-kb downstream of the *Ds* insertion. To check the enhancer effect, we performed semi-quantitative PCR analysis of DS-16 and WT root and leaf tissues and the result showed the activation of only LOC_Os01g08790 gene, which was expressed in both root and leaf tissues whereas its expression was not detected in WT (Fig. 2B). At the same time, we also checked the 4X enhancer effect on other three neighboring genes. We did not detect any expression in all the three genes (LOC_Os01g08800, LOC_Os01g08810 and LOC_Os01g08814) (data not shown). Further, we analyzed the transcript level of LOC_Os01g08790 by a real-time analysis and observed higher expression (8-fold) of LOC_Os01g08790 gene during control condition in the leaves of DS-16 with respect to WT. However, the level of transcript was enhanced 100-fold during NaCl stress treatment (Fig. 2C) as compared to WT. Similarly, in roots, it was observed also under control condition with the transcript level being quite low but with a little increased (1.5-fold) transcript level during salt stress treatment (Fig. 2D). At the same time, we checked transcript levels of the other upstream genes (LOC_Os01g08800, LOC_Os01g08810 and LOC_Os01g08814), which showed no detectable expression pattern in both leaf and root tissues of DS-16 plant (data not shown). Therefore, only one gene at the locus, LOC_Os01g08790 in DS-16 plant was activated by CaMV35S 4X enhancer elements, which also showed higher expression during abiotic stress treatments, particularly salt stress in this study.

**Figure 3:**
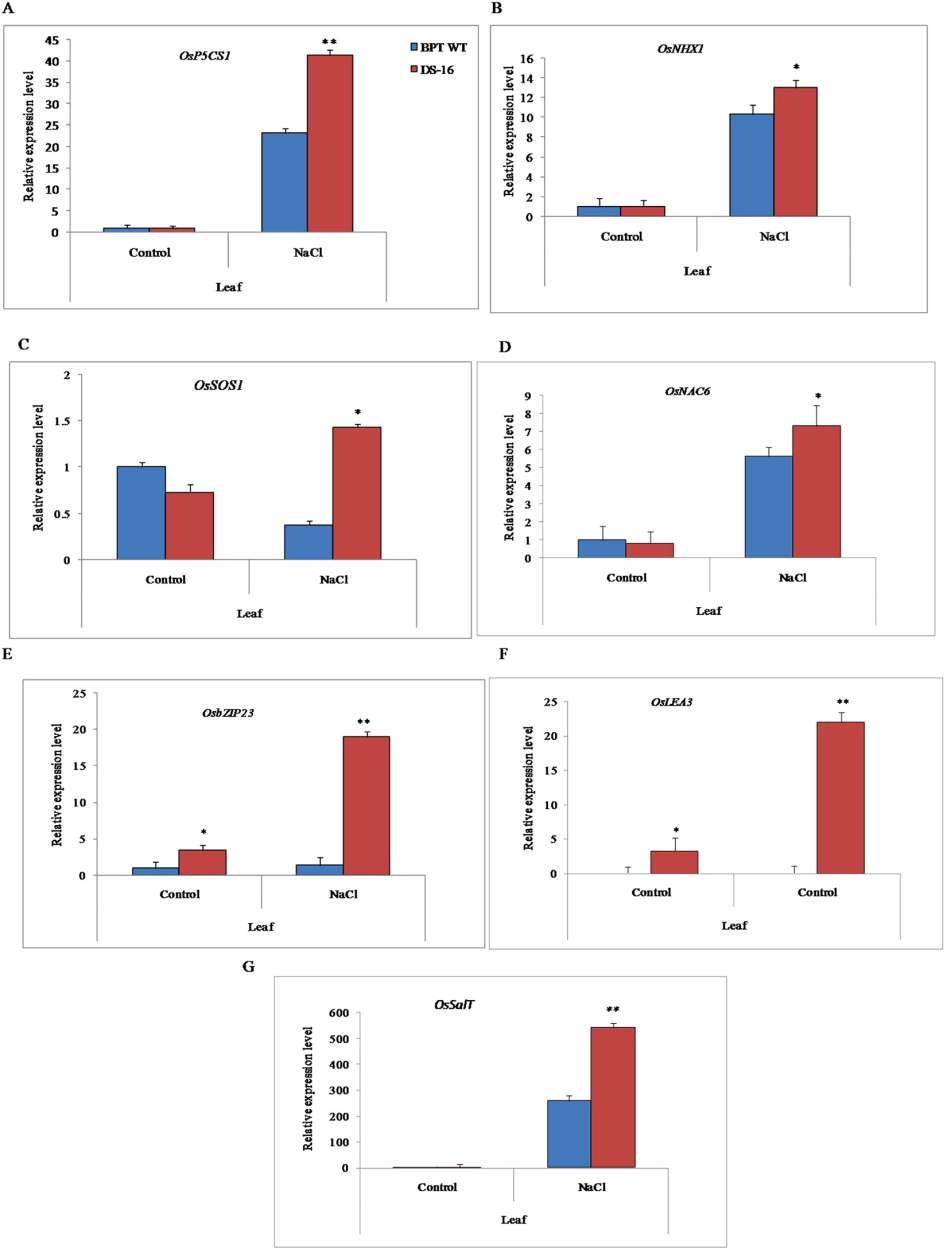
Analysis of transcript levels of stress responsive marker genes in activation tagged DS-16 line in comparison with WT-BPT under NaCl stress treatment. (**A**) *OsP5CS1*; (**B**) *OsNHX1*; (**C**) *OsSOS1*; (**D**) *OsNAC6*; (**E**) *OsbZIP23*; (**F**) *OsLEA3*; (**G**) *OsSalT*. Two weeks old seedlings grown in Yoshida’s solution supplemented with 150 mM NaCl for 15 d and those without NaCl supplement used as control. *OsActin1* was used as a reference gene for quantitative real-time RT-PCR. Means of three independent samples and standard errors are presented. ** and * indicates significant difference at P < 0.01 and P < 0.05.

Since we have observed enhanced expression of tagged gene during NaCl treatment, we analyzed the expression of a few selected stress-responsive genes such as *OsP5CS1, OsNAC6, OsLEA3, OsSOS1, OsZIP23, OsNHX1* and *OsSalT* in the leaf tissues of DS-16. Interestingly, quantitative analysis revealed a significantly enhanced expression of these genes under NaCl stress as compared to the WT and as well as under control conditions (Fig. 3A–G). Though there was some up-regulation of some of these genes in the stressed WT plants also, the expression particularly of *OsSOS1*, *OsbZIP23* and *OsSalT* was significantly higher. The results clearly suggested that the tagged gene, LOC_Os01g08790 has a role in salt stress conditions.

### LOC_Os01g08790 identified as *OsNF-YC13* and phylogenetic analysis of the gene

Previous reports showed that there are 28 NF-Y genes identified in the rice genome^34, 43^. However, a recent report included some new genes as new *OsNF-Y* genes. As a result, 40 putative NF-Y genes in rice were annotated, which included 11 NF-YA, 13 NF-YB, and 16 NF-YC^44^. We searched gene at the locus LOC_Os01g08790 that got activation-tagged in the present investigation in the Plant Transcription factor database (PlantTFDB) (http://planttfdb.cbi.pku.edu.cn). Apart from the earlier annotated 7 NF-YC transcription factors, 5 new members viz., *OsNF-YC9* to *OsNF-YC12* ^48^ were included in the PlantTFDB. We found the tagged gene LOC_Os01g08790 in the PlantTFDB and the protein has been predicted to possess a coiled coil region (from 3 to 47 AA) and NF-YC domain (from 119 to 180 AA) with a score of 45.4 and 1.8e-14 E-value (Supplementary Fig. 3A). Hence, we named LOC_Os01g08790 as *OsNF-YC13*. The *OsNF-YC13* gene has an uninterrupted ORF of 717 bp, encoding a 239-amino acid protein with the polypeptide mass of 25.146 KDa and an isoelectric point (pI) of 4.21.

To construct the phylogenetic tree, a total of 16 unique NF-YCs from the *O. sativa* genome (http://planttfdb.cbi.pku.edu.cn) were retrieved for performing the BLASTp analysis. The *OsNF-YC13* showed similarity to TFs present in the wild rice genotypes. Further, the phylogenetic analysis of all the members of NF-YCs family in *O. sativa* with those of the wild rice accessions, *S.bicolor*, *S.italica* and *G.max* indicated that *OsNF-YC13* is separately grouped to the TFs of wild rice and other monocot species (Supplementary Fig. 3B). Multiple sequence alignment of *OsNF-YC13* with the wild rice accessions indicated a 100% identity with NF-YC TFs of *O.rufipogan*, 99% with *O.nivara*, 56% with *Sorghum bicolor* and 55% with *Setaria italica* and very less homology with the members of *A.thaliana* (20%) and *Glycine max* (17%) (Supplementary Fig. 3C and Supplementary Table 3).

### Expression of *OsNF-YC13* under salt stress

**Figure 4:**
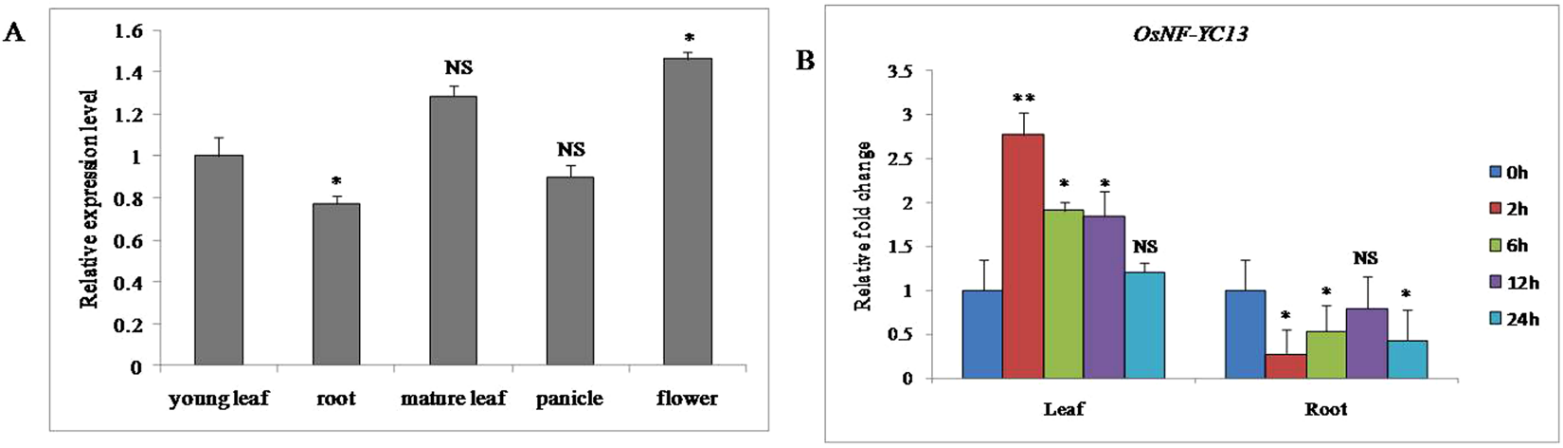
Expression pattern of OsNF-YC13 in wild-type BPT 5204. (**A**) Relative expression profile of OsNF-YC13 gene in different organs of wild-type rice plants. The transcript level of OsNY-YC13 in young leaf as standard. (**B**) Relative expression of OsNF-YC13 under NaCl stress (150 mM) treatment in leaf and root. Two week-old rice seedlings grown in Yoshida’s solution were collected for gene expression analysis of different time intervals. Total RNA was prepared from 2-week-old seedlings of wild-type rice after the above treatments and then cDNA was prepared. Real-time PCR was performed using *OsActin1* was used as an internal control. qRT-PCR was performed with *OsNF-YC13* specific primers. Data represent means and standard errors of three biological replicates are shown.

The transcripts of *OsNF-YC13* were detected in different organs with higher level in mature leaf and flower of control WT-BPT (Fig. 4A). The response of *OsNF-YC13* expression under NaCl (150 mM) was monitored in a real time analysis. The *OsNF-YC13* transcripts in shoot got gradually decreased after 6–24 h of NaCl treatment respectively (Fig. 4B). Similarly, we observed the expression pattern of *OsNF-YC13* in roots where the transcript level showed a ≤ 2-fold change in NaCl stress (Fig. 4B). The result indicated that *OsNF-YC13* transcription factor was differentially regulated in salt stress treatments with shoot specific expression.

### Subcellular localization of *OsNF-YC13*

**Figure 5:**
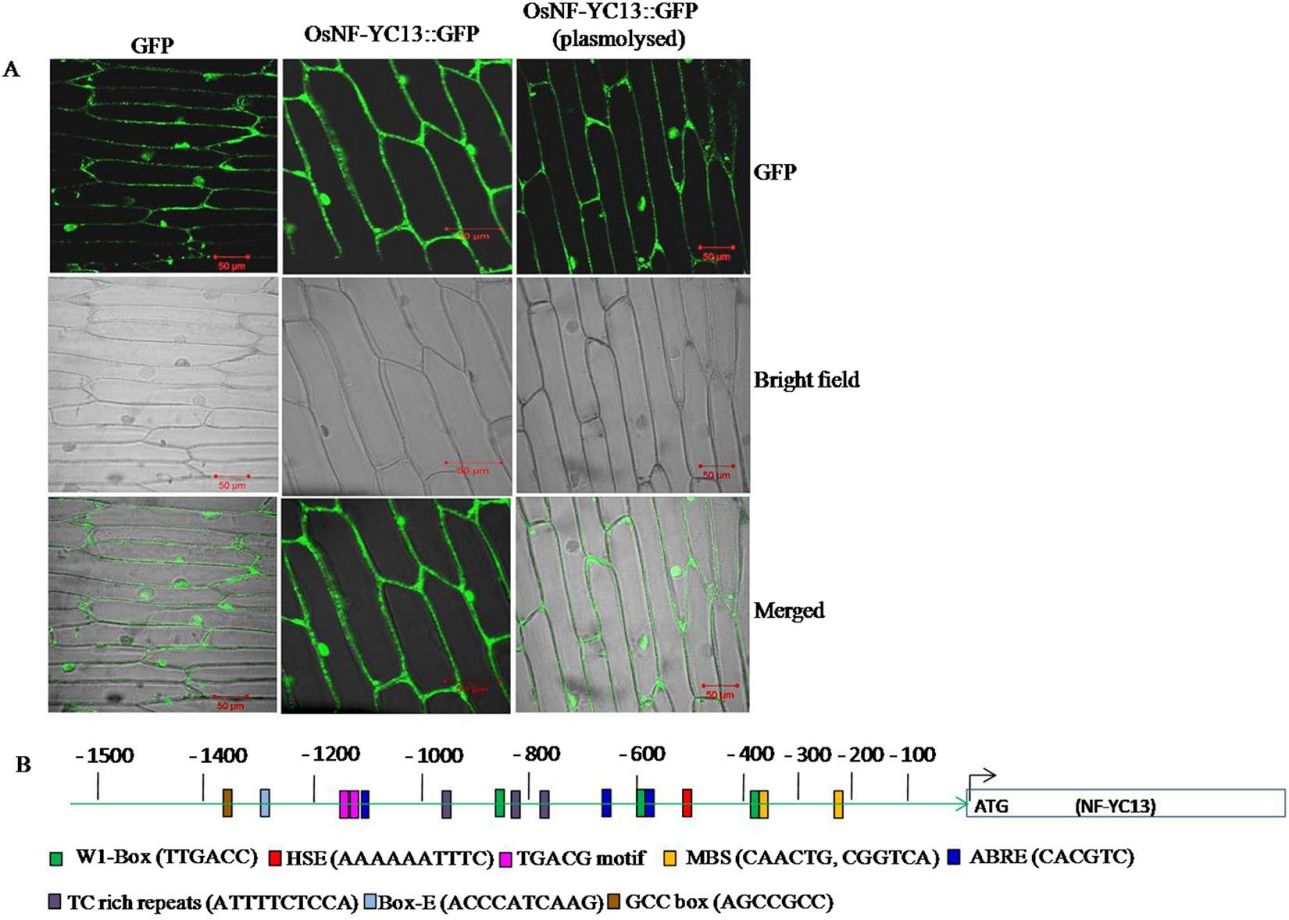
Subcellular localization of OsNF-YC13 in onion epidermal cells and graphical representation of various *cis*-regulatory elements in 1.5 kb upstream regulatory region of *OsNF-YC13*. (**A**) The coding region of *OsNF-YC13* was fused to the N-terminus of green fluorescence protein (GFP) and transformed into onion epidermal cells through *Agrobacterium*-infiltration using GFP as control. The GFP fluorescence was detected under confocal laser scanning microscope. (**B**) Physical mapping of key *cis-*elements involved in different stresses. A 1.5 kb upstream region of *OsNF-YC13* was extracted from rice genome database and searched for various stress related elementsusing online tool, PlantCare.

To determine the subcellular localization of the *OsNF-YC13*, we generated *OsNF-YC13*::GFP fusion constructs under the control of CaMV 35S promoter. These constructs were then expressed in the onion epidermal cells. We observed that *OsNF-YC13*::GFP protein fluorescence was localized to the nucleus and in the peripheral region of the transformed onion cells (Fig. 5A). Further, the transformed onion cells were subjected to plasmolysis, which showed that the GFP was present in nucleus as well as cytoplasm (Fig. 5A).

### Yeast transactivation assay of OsNF-YC13 and possible interacting proteins

To check the transactivation activity of OsNF-YC13 subunit, we performed transcriptional activation assay using yeast cells showed that OsNF-YC13 did not possess transactivational activity alone (Supplementary Fig. 4A). In order to find out the possible other protein interaction network with OsNF-YC13, we used STRING database (https://string-db.org/). The result showed that OsNF-YC13 might have interacted with OsNF-Y B subunits such as NF-YB3, B4, B6, B7 and others (Supplementary Fig. 4B).

### *Cis*-elements in 1.5-kb promoter region of *OsNF-YC13*

Because of the up-regulation of *OsNF-YC13* gene in other abiotic stresses, we investigated for the presence of stress responsive *cis*-elements in the putative promoter region upstream to the coding region. We extracted 1500-bp upstream sequence from rice database and analyzed using PlantCARE database^49^. We found various stress responsive *cis*-elements in the 1.5 kb region, which were physically mapped (Fig. 5B). Major stress-responsive *cis*-elements such as abscisic acid responsive elements (1 ABRE: CACGTC), heat stress elements (1 HSE: AAAAAATTTC), low temperature responsive elements (2 LTR: CCGAAA), MYB binding site (2 MBS: CAACTG, CGGTCA) which is involved in drought-inducibility, methyl jasmonate responsive elements (2 TGACG motif, 1 CGTCA motif), TC-rich repeats (2 ATTTTCTCCA) involved in defense and stress reponse, GCC box (1 AGCCGCC), Box-W1 motif (3 TTGACC), which are responsive to fungal elicitors were found in the 1.5-kb upstream regulatory region.

### Increased growth and biomass of activation-tagged line DS-16

**Figure 6:**
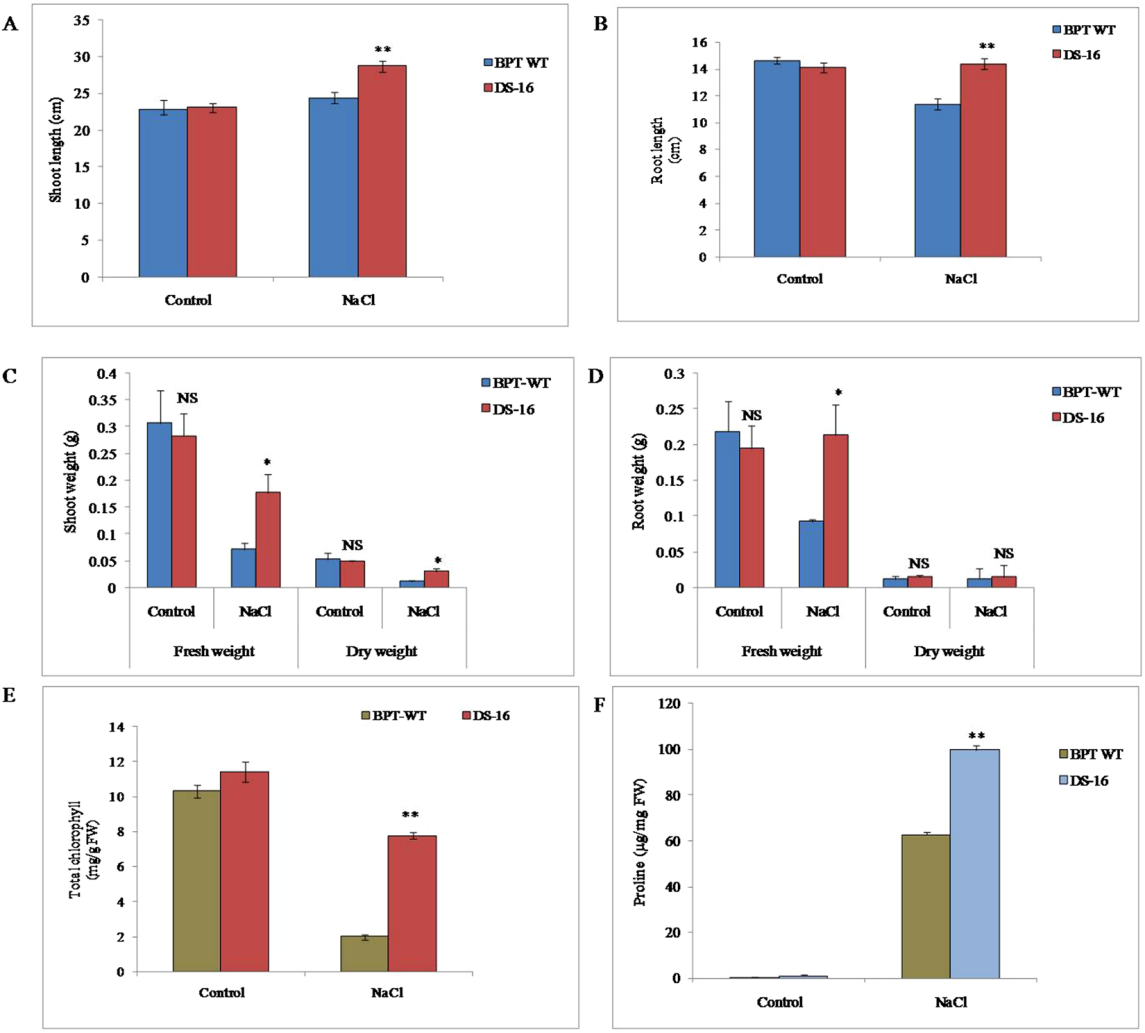
Evaluation of activation tagged DS-16 plants for salinity tolerance and chlorophyll and proline content in the activation tagged DS-16 line. (**A**,**B**) Shoot and root length. (**C**,**D**) Fresh and dry weight of shoot and root. E: Total chlorophyll content (**F**) proline content. Two weeks old rice seedlings from DS-16 and WT-BPT were treated with 150 mM NaCl stress treatment for 2-weeks. Experiment was carried out before and after stress treatment of DS-16 and WT. A total of 5–10 rice seedlings were measured and two independent biological repeats were performed for each treatment with error bar. ** indicates significant differences in comparison with the WT at P < 0.01, *P < 0.05, NS - non significant.

The seedling growth and biomass were measured before and after NaCl stress treatments in the salt tolerant DS-16 line. The shoot and root length of both WT and DS-16 were similar in length during control condition whereas both shoot and root length were increased significantly as compared to that of WT seedlings under sodium chloride stress (Fig. 6A and B).

Similarly, the weight of the plants has not been affected by salt stress. Fresh and dry weight of DS-16 shoots under salt stress treatment was significantly increased compared with that of WT. However, no significant changes were observed in both the tagged line and WT without stress. In addition to shoot biomass, root biomass was also measured after stress treatment (Fig. 6C). During stress condition, the root fresh weight of WT was negatively affected and decreased by 10% compared to its control condition. But, in the tagged line, the root fresh weight was not affected during NaCl stress condition. With no change in the fresh root mass of DS-16, the dry weight of tagged line was on par with WT root (Fig. 6D). Overall, the tagged line DS-16 was appeared to be tolerant to 150 mM NaCl stress treatment compared with the WT.

### Estimation of total chlorophyll and proline content

Leaf of DS-16 and WT was collected freshly before and after NaCl stress treatment for measuring the total chlorophyll and proline contents. Under control condition, there were no significant differences in the chlorophyll content between WT and DS-16 plants. However, after two weeks of NaCl stress condition, the chlorophyll content of WT plants was significantly lower than that of DS-16. The mean chlorophyll contents of DS-16 plants were found a 3.8-fold higher (7.7 mg g^−1^FW) under NaCl stress as compared with WT (2.0 mg g^−1^ FW) (Fig. 6E). In addition to total chlorophyll content estimation, proline content was also measured. Under control condition, there were no significant differences in proline content between DS-16 and WT. However, after two weeks of NaCl stress treatment (150 mM), the proline content in DS-16 and WT plants was measured as 99.8 µg mg^−1^ FW and 62.6 µg mg^−1^ FW respectively. A 1.5-fold elevated proline content was observed in DS-16 plants as compared to the WT (Fig. 6F). The results of higher chlorophyll and proline content in DS-16 are consistent with the salt tolerance phenotype in DS-16.

### Accumulation of Na^+^ and K^+^ content in leaf and root of DS-16 during salt stress

**Figure 7:**
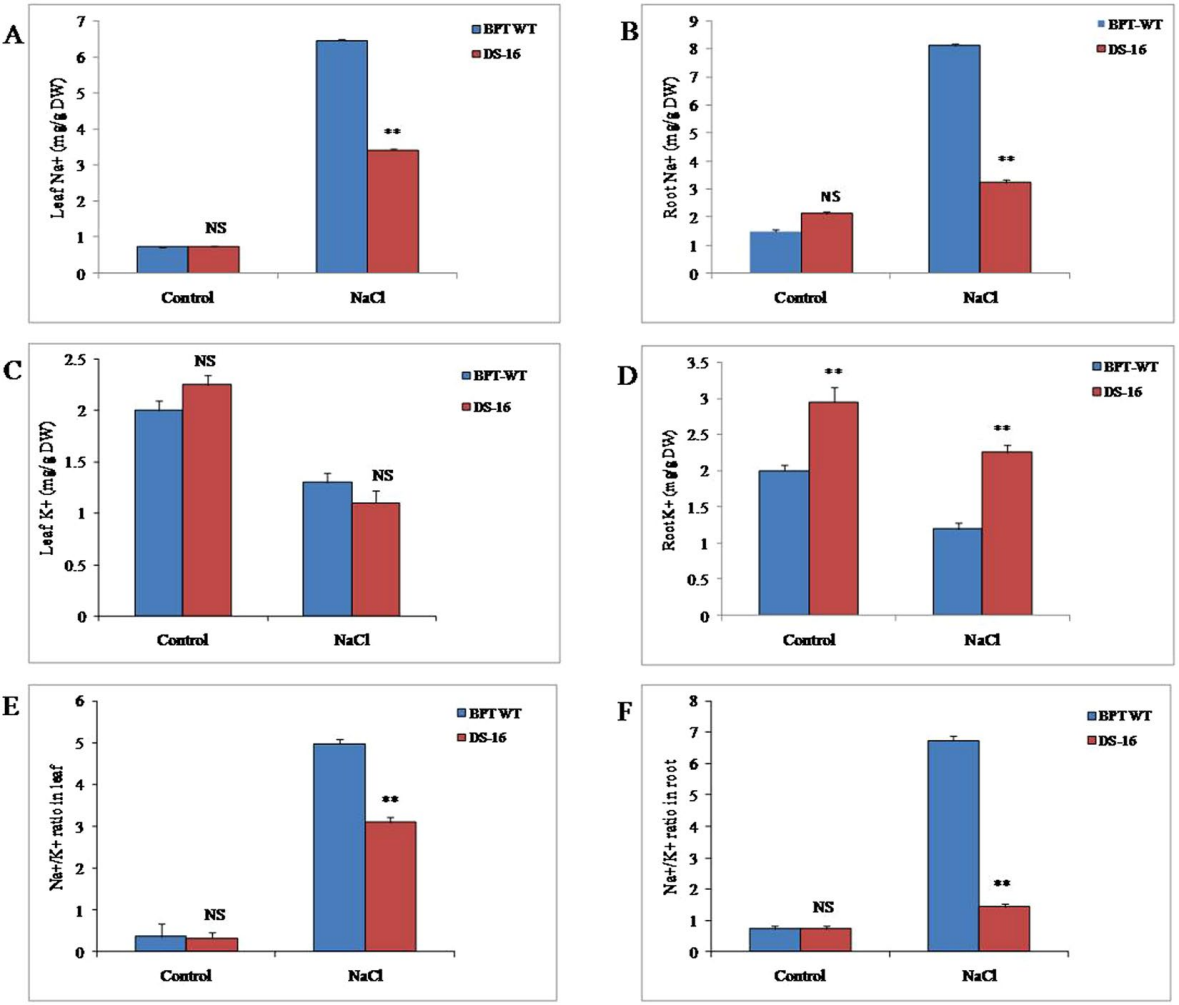
Estimation of Sodium and potassiumcontent of salt tolerant activation tagged DS-16 line. (**A**) Leaf Na^+^, (**B**) root Na^+^, (**C**) leaf K^+^, (**D**) root K^+^, (**E**) Leaf Na^+^/K^+^ ratio, (**F**) root Na^+^/K^+^ ratio. Two weeks old rice seedlings from DS-16 and WT-BPT were treated with 150 mM NaCl stress for two weeks. Ion accumulation was estimated before and after stress treatment in DS-16 and WT. Three rice seedlings were used in each treatment and three independent biological repeats were performed for each treatment with error bar. * indicates significant differences in comparison with the WT at P < 0.05, **P < 0.01, NS - non significant.

Accumulation of sodium and potassium ions in the seedlings of DS-16 and WT under control and NaCl stress conditions was investigated. As shown in Fig. 7A, the Na^+^ concentration in leaves of DS-16 and WT control plants showed no significant changes under control condition. In contrast, the concentration of Na^+^ in leaves of WT increased by ~9-fold under 150 mM NaCl stress (from 0.74 to 6.46 mg g^−1^ DW) in comparison with its control conditions (no stress). At the same time, the DS-16 line showed only a five-fold increase in Na^+^ concentration (from 0.74 to 3.41 mg g^−1^ DW) compared to its control condition. Relatively, the DS-16 plants maintained a ~1.8-fold lower Na^+^ concentration under NaCl stress conditions compared to the WT. Similarly, under control and NaCl stress conditions, no significant changes in K^+^ accumulation were observed in both WT and DS-16 (Fig. 7C).

Further, we measured the Na^+^ and K^+^ concentrations in the roots. There were no significant differences in the accumulation of Na^+^ in roots in both types in the control condition. However, NaCl stress resulted in a 2.5-fold decreased Na^+^ in roots of DS-16 plants compared to WT (Fig. 7B). Besides the presence of a lower level of Na^+^ in DS-16, a 1.4- and 1.8-fold higher K^+^ accumulation was observed in the roots of DS-16 during control and NaCl stress conditions respectively compared to the WT (Fig. 7D). Further, we calculated Na^+^/K^+^ ratio in leaf and root of DS-16 and WT control under both conditions. During control condition, no significant differences were observed in the ratio in leaf and root of both types of plants, whereas under NaCl stress, the ratio decreased to 1.5-fold in leaf and 4.7-fold in root of DS-16 as compared with that of WT (Fig. 7E and F). This analysis clearly indicated that the DS-16 line retained lower Na^+^ and higher K^+^ concentration in roots and lower Na^+^ in leaf under salt stress conditions for maintaining ion homeostasis.

### Analysis of antioxidant enzyme activity

**Figure 8:**
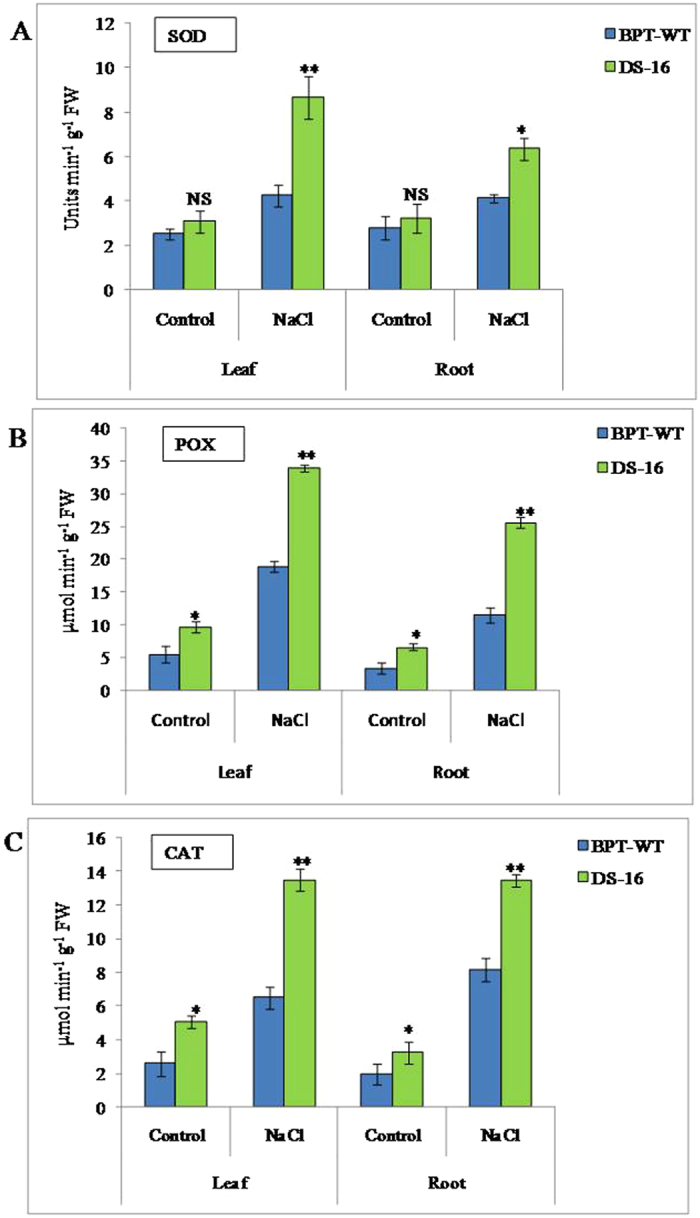
Analysis of antioxidant enzymes activity in activation tagged DS-16 and WT before and after NaCl treatment. (**A**) super oxide dismutase (SOD), (**B**) peroxidase (POX), (**C**) catalase (CAT). Two weeks old DS-16 seedlings and WT-BPT were treated with 150 mM NaCl for twoweeks. Three rice seedlings were used in each treatment and three independent biological repeats were performed for each treatment with error bar. * indicates significant differences in comparison with the WT at P < 0.05, **P < 0.01, NS - non significant.

Antioxidant enzymes such as superoxide dismutase (SOD), peroxidase (POX) and catalase (CAT) are involved in scavenging ROS and protecting the cells from the oxidative damage and eventually leading to enhanced stress tolerance. In the present study, these three enzymes were analyzed at the seedling stage and they were significantly increased before and after NaCl stress in DS-16 plants as compared to WT. SOD activity increased1.5-fold under NaCl stress conditions in both shoot and root of DS-16 plants as compared with that of WT (Fig. 8A). Similarly, enhanced POX (1.7-fold) and CAT (2-fold) activities were observed in shoots of DS-16 plants (Fig. 8B and C), while in the root, the POX and CAT activity increased by a 2.2- and a 1.6-fold respectively (Fig. 8B and C). The increased content of SOD, POX and CAT would result in reduced oxidative damage to cells caused by salt stress treatment and enhances tolerance in DS-16 to 150 mM NaCl stress.

## Discussion

Plants are constantly exposed to various abiotic stresses causing yield losses. Salinity is one of the major abiotic stresses that reduces the plant growth and production especially in food crops^50^. To reduce the yield loss by increasing soil salinization, breeders have developed various salt-tolerant rice varieties by introgressing QTL/genes conferring salt tolerance from other related genetic resources and subsequent identification based on marker-assisted breeding. On the other hand, deploying the novel gene(s) or altering the expression of existing genes to increase the degree of salt tolerance by generation of transgenic plants is an option that needs thorough investigations^51^.

Rice (*Oryza sativa* L.) crop is more sensitive to increased soil salinization at seedling and reproductive stages^52, 53^. Development of salinity tolerant rice varieties is an efficient option to overcome salinity stress and enhance productivity under stress. Activation-tagging (AT) system has been recently used for developing gain-of-function mutants in a widely cultivated *indica* rice variety (BPT 5204, Samba Mahsuri) to identify novel genes^20^. Many research groups utilized this system earlier for developing large mutant populations in different plants species (Arabidopsis, *japonica* type Rice, Tomato, Potato, Strawberry, Poplar, Barley, Sorghum) as resources for functional genomics.

In the present study, we have screened activation-tagged *indica* rice lines on 150 mM sodium chloride and developed an activation-tagged rice line showing enhanced expression of a previously uncharacterized gene, LOC_Os01g08790 under salt stress treatment. Further, the phenotype of tagged salt tolerant line was characterized by physiological, biochemical and molecular expression analyses. Sequence analysis of the corresponding activation-tagged loci in selected salt tolerant rice *Ds* plants revealed that the integration of the *Ds*-Enhancer elements was in intragenic region (95%) of most of the plants screened and the remaining (5%) were in the intergenic regions on the chromosomes. This result was consistent with other earlier reports where most of the insertions were maximally in intragenic (coding region, promoter, 3′ UTR), while it was lesser in intergenic regions^22, 54^. From the twenty salt tolerant *Ds* plants, DS-16 was selected for further analysis because the tagged gene was intergenic, with a novel/uncharacterized gene encoding ‘Histone-like transcription factor’ (LOC_Os01g08790) getting activated by the CaMV 35S 4X enhancers during NaCl stress conditions. Expression analysis of DS-16 plant by qRT-PCR revealed that the tagged gene histone-like transcription factor (LOC_Os01g08790), where the *Ds*-4X Enhancer positioned between LOC_Os01g08790 and LOC_Os01g08800 (Cytochrome P450) was expressed at a 8-fold enhanced level under control condition while the transcript level was enhanced to a 100-fold during NaCl stressed condition.

Previous reports suggested that one or two genes can be activated at a time by the multiple CaMV enhancers in different *Ds* lines^20, 47^. It has also been reported that the degree of activation and distance of the *Ds* insertion to the gene has no correlation with the enhancer function^16, 47^. In our investigations on the DS-16 line, only one gene (LOC_Os01g08790) was activated by the CaMV 35S 4X enhancers and the transcript level was elevated during salt stressed condition. As the LOC_Os01g08790 gene showed higher transcript level during NaCl stress condition, we analyzed the expression level of a few selected stress-responsive genes (*OsP5CS1, OsNAC6, OsLEA3, OsSOS1, OsZIP23, OsNHX1* and *OsSalT*) based on the previous reports, which established that these stress-responsive genes were up-regulated during abiotic stress condition particularly in salinity stress^55, 56^. Interestingly, these stress-responsive genes were also up-regulated in DS-16 plants with significantly higher expression level during salt stressed conditions and also associated with significantly up-regulated LOC_Os01g08790 gene expression. Theses result indicates that the gene LOC_Os01g08790 has a significant role in abiotic stress condition, particularly in salt stress.

The newly identified gene LOC_Os01g08790 encodes a putative histone-like transcription factor that belongs to nuclear factor Y-C subfamily and we named it as *OsNF-YC13* with the data presented in the Results section. The Nuclear factor Y (NF-Y), is a heterotrimeric complex transcription factor consisting of three subunits, namely NF-YA (also called HAP2 or CBF-B), NF-YB (HAP3 or CBF-A), and NF-YC (HAP5 or CBF-C)^43^. A total of 40 putative NF-Y genes including 11 NF-YAs, 13 NF-YBs and 16 NF-YCs were found in rice database^44^.

Further, we have studied the localization of the transcription factor activity through transient expression of *OsNFYC13*::GFP fusion protein construct in transformed onion epidermal cells, which indicated that the gene is localized to nucleus and cytoplasm, which is consistent with the previous reports on *CdtNF*-YC1 from Bermudagrass^57^ and *OsNF-YB1* ^58^. Localization of *OsNF-YC13*::GFP in both nucleus and cytoplasm of onion cells suggests that the NF-YC13 may interact with other unknown OsNF-YB subunits to from dimers that translocate from cytoplasm to nucleus. Similar to our result, localization of *OsNF-YB1*::GFP was observed in both nucleus and cytoplasm of root cells whereas the protein confined to nucleus of aleurone layers cells^58^. In the present study, we could not observe transcriptional activation of OsNF-YC13 alone as no colonies were observed on the selection medium, which indicate transactivation. The gene might require additional protein interactions to regulate transactivation and modulate the expression of downstream targets. Similar to our result, Xu and co-workers^58^ found no transcriptional activation of OsNF-YB1 alone and it requires additional partner like OsERF#115 factor. Analysis using the STRING online tool showed that the NF-YC13 might interact with NF-YB subunits B3, B4, B6, B7 etc.

NF-Ys have diverse functions in plant growth, development and also in stress responses^34, 59^. Several groups worked on different individual NF-Ys in model systems and characterized their role in development and physiological process^34, 46^. For instance, one of the reports demonstrated that *AtNF-YB9* (LEC1) plays a key role in embryo development^60^. Similarly, a rice nuclear factor Y, *OsNF-YB1* is specifically expressed in the aleurone layer of developing endosperm regulating grain filling and endosperm development^58^. Zhu and co-workers^61^ identified a new uncharacterized gene *OsHAPL1* (NF-YC like) with involvement in spikelet heading date in rice. After characterization, they proposed that *OsHAPL1* functions as a transcriptional regulator and forms a complex with DTH8, Hd1, which influences the heading date in rice^61^. The above reports suggest that NF-Y proteins play a crucial role in plant developmental process.

Although NF-Y proteins play crucial roles in plant development, they also appear to play roles in biotic/abiotic stresses. Recently, Chen and co-workers^57^ have investigated the expression profile of some rice NF-YC genes under dehydration, salt and ABA stress treatments. Genome wide expression analysis of soybean with 68 NF-Y (21 GmNF-YA, 32 GmNF-YB, 15 GmNF-YC and 13 NC2) genes demonstrated their differential expression in response to drought stress^39^. In our case, the identified *OsNF-YC13* gene was differentially regulated in salt stress (NaCl) treatment.

Many individual NF-Y subfamily genes were characterized separately through transgenic approach and they appear to be involved in different plant physiological process including abiotic stress condition. Overexpression of *AtNF-YB1* in *Arabidopsis* and maize (*Zea mays*) was shown to significantly improve drought resistance and yield under drought stress conditions^62^. Overexpression of *AtNF*-*YA5* increased drought tolerance in Arabidopsis plants^63^. Similarly, overexpression of *OsNF-YA2* (*OsHAP2E*) in rice also showed resistance to *Magnaporthe oryzae or Xanthomonas oryzae pv. oryzae* with tolerance to salinity and drought^64^. Ectopic expression of Bermudagrass NF-YC (*Cdt-NF-YC1*) in rice showed elevated tolerance level to drought, salt and sensitive to ABA^57^. Overexpression of garlic NF-YC (*AsNF-YC8*) in tobacco conferred tolerance to drought and salinity stress^65^.

Since the *OsNF-YC13* was induced during salt stress condition, we tried to focus on various stress responsive *cis*-regulatory elements such as ABRE, HSE, LTR, MBS, methyl jasmonate responsive elements, TC-rich repeats, GCC box, Box-W1 motif in the 1.5 kb putative promoter region of *OsNF-YC13*. The presence of various stress responsive elements in the promoter region of the gene supported our observations on the enhanced expression of the novel *NF-YC13* in rice under abiotic stress particularly salinity condition. As the stress responsive genes were up-regulated during NaCl treatment in DS-16 plants, we searched for the presence of CCAAT elements in the promoter region of *OsP5CS1*, *OsNHX1*, *OsLEA3*, *OsbZIP23*, *OsSOS1* and *OsSalT* genes. Interestingly, we observed the presence of 1–2 CCAAT *cis*-elements in the promoters of all the genes except *OsNHX1* and *OsbZIP23* (Supplementary Table 4). Similar to our results, Alam *et al*.^64^ found 1–5 CCAAT *cis*-elements in the promoter regions of ABA induced marker genes that got up-regulated under salt and drought stress treatments in rice transformed with *Cdt-NF-YC1*. The up-regulation of selected stress responsive genes in DS-16 plants correlated with the presence of respective stress responsive elements in the promoters of most the stress related genes suggests that *OsNF-YC13* holds a role during salt stress condition through directly or indirectly activating the ABA-dependent signaling pathway.

Because of *OsNF-YC13* gene was induced during NaCl treatment, the DS-16 was further characterized for salinity tolerance by using physiological and biochemical analyses. As rice seedlings are very sensitive to salinity stress, the seedlings (15 d old) of DS-16 after salt stress treatment showed significantly better growth (shoot and root length), biomass and higher chlorophyll content than its WT. Our result was consistent with the over-expression of *OsNF-YA2* (*OsHAP2E*) in rice, which resulted in enhanced shoot, root length, weight and higher chlorophyll content under salinity treatment^64^. Increased accumulation of free proline in plants has been shown increase plant tolerance to osmotic stress and the free proline not only acts as free radical scavenger but also protects from cell damage during stress condition^66^. Also DS-16 showed significantly higher free proline content accumulation compared to the WT under NaCl stress treatment.

Plants exposed to high salinity experience both ionic and osmotic stresses, which restrict Na^+^ uptake and entry into their cells. Increased Na^+^ levels are toxic to cells and as the Na^+^ ion competes for binding sites with K^+^ as it has similar physiochemical properties and this leads to metabolic disturbances causing severe cellular damage^67, 68^. Thus, plants either adapt to or evade salt stress by excluding Na^+^ from the roots thereby maintaining a lower level of Na^+^ in the leaves^69^. Salt-tolerant plants accumulate salt in the vacuoles thereby controlling lower salt concentration and maintaining higher K^+^/Na^+^ ratio in the cytoplasm of the cells^70^. There are several studies reporting that transgenic or salt-tolerant plants accumulate less Na^+^ and more K^+^ maintaining a lower Na^+^/K^+^ ratio than the control or susceptible plants^52, 71, 72^. Consistent with these reports, DS-16 plants showed significantly lower level of Na^+^ in leaves and roots under salt stress condition whereas no significant accumulation level of K^+^ was observed in leaves. However, the roots of DS-16 plants accumulated higher level of K^+^ accumulation than its WT control. As a result of this, they maintained lower level of Na^+^/K^+^ ratio in leaves and roots with better ion homeostasis that prevents cell damage.

During abiotic stress condition, plants should maintain low level of reactive oxygen species (ROS) to minimize the cellular damage caused by osmotic stress^73^. Plant defense system possesses ROS-detoxification antioxidant enzymes like SOD, CAT and POX for scavenging ROS and reduced oxidative damage^74^. In our study, we have checked the antioxidant enzyme activities in DS-16 plants, which showed higher activities of SOD, POX and CAT in comparison with WT under salt stress treatment. Evidence supports reduced oxidative damage and increased salinity tolerance in plants are associated with efficient antioxidant defence system in plants^75, 76^. Similarly, overexpression of garlic NF-YC, (*AsNF-YC8*) in tobacco showed higher antioxidant enzymes activity (SOD, POX, CAT, APX) than the wild type tobacco^65^.

Overall, with the results from above experiments, we identified an activation-tagged line DS-16 for salt tolerance at 150 mM NaCl and studied the expression of a novel and previously uncharacterized gene *OsNF-YC13*, which was identified a candidate gene for salt tolerance. This novel gene was induced under different abiotic stress conditions. The activation-tagged DS-16 plants exhibited salinity tolerance, which was supported by physiological, biochemical and expression analysis of several stress responsive genes. From this present study, we conclude that the novel *OsNF-YC13* gene has a significant role in salinity stress in rice. Further studies are being made in rice to demonstrate its role during plant developmental/physiological process.

## Materials and Methods

### Plant material, seed germination and salt stress treatment

Seeds of rice plants in T_3_ generation carrying both *Ac* and *Ds* (with multiple 35S enhancers) elements and wild-type BPT 5204 (WT) were surface sterilized with 70% ethanol and 0.1% HgCl_2_ solution. The sterilized mature seeds were inoculated on hormone free ½ MS basal medium. The plates were incubated under light for 15 d for germination. Two week old transgenic rice seedlings were directly transferred to Yoshida’s culture solution^77^ for 3–4 d followed by salt stress treatment in Yoshida’s solution supplemented with 150 mM NaCl (pH 5.8) for two weeks. The solution was prepared freshly and replaced after every three days to maintain pH 5.8. Two week after salt stress treatment, the tolerant transgenic rice plants were recovered and transplanted to pots and maintained in the green house for further characterization.

To examine the expression pattern of *OsNF-YC13* gene, which got activated by CaMV 35S 4X enhancers in salt stress treatment, two week old rice seedlings (WT) were transferred to Yoshida’s culture solution supplemented with sodium chloride (NaCl; 150 mM) for salt treatment. After treatment, shoot and root samples were collected separately at 2 h, 6 h, 12 h and 24 h intervals. Treatment with water served as non-stressed control (0 h). RNA isolation and gene expression analysis under various stress treatments was performed using qRT-PCR.

### PCR confirmation of salt tolerant activation-tagged rice transgenic plants

The DNA samples isolated from leaves of salt tolerant rice mutants and non-transformed control plants were used in PCR analyses^78^. PCR amplification was performed with 50 ng of template DNA, using primers for *hph* (hygromycin phospho-transferase) and *RFP* (Red fluorescent protein)-*Nos* (nopaline synthase termination signal) using a Thermal Cycler (Eppendorf, Germany). The PCR reaction mixture (10 μL) was prepared with genomic DNA, 1 × Taq assay buffer (containing 1.5 mmol/L MgCl_2_), 125 μmol/L dNTPs, 0.4 μmol/L each of forward and reverse primers and 1 U of Taq DNA polymerase (KAPA Biosystem, USA). The PCR profile was set as follows: 95 °C for 5 min, 95 °C for 30 s + 58 °C for 30 s + 72 °C for 1 min (35 cycles) + 72 °C for 7 min. The PCR products were resolved on 1% TAE agarose gels. Gene specific primers used in this study are listed in Supplementary Table 1.

### Identification of DS insertion site by TAIL-PCR and flanking sequence analysis

Thermal Asymmetric Interlaced Polymerase Chain Reaction (TAIL - PCR)^79^ was performed using Ex-Taq DNA polymerase (Takara, Japan) in Eppendrof thermal cycler (Eppendrof, Germany) following Moin *et al*.^20^. Genomic DNA isolated from salt tolerant rice plants was subjected to three separate PCR runs using a degenerate primer and three nested primers specific for the 5′ end of the *Ds* element for the identification of the insertion in the rice genome and analysis of flanking sequences. The details of degenerate and nested primers have been detailed in earlier report^20^. The final products were run on 1% agarose gels and the expected products were sliced out for purification and sequencing. Sequences were checked using the BLAST against the rice annotation sites at MSU (http://rice.plantbiology.msu.edu), TIGR (http://blast.jcvi.org/euk-blast/) and RAP-DB (http://rapdb.dna.affrc.go.jp/).

### RNA isolation and Real-time PCR analysis of activation-tagged line

Total RNA from leaf and root tissues of activation-tagged transgenic and wild-type BPT 5204 (both control and stressed conditions) was isolated according to the manufacturer’s instructions (RNAiso Plus, Takara, Japan) and PCR was performed as per Manimaran *et al*.^80^. Two microgram of total RNA was used for first-strand cDNA synthesis using oligo d(T) primers (SMART MMLV RT, Takara, Japan). The cDNA after RNaseH treatment was mixed with 12.5 µL of 2X KAPA SYBR® FAST qPCR mix universal (KAPA Biosystem, USA), 2 µM each of forward and reverse primers in a final volume of 20 µL. PCR with no template control (NTC) was also performed for each primer pair. The real-time PCR was performed in Mastercycler® ep realplex (Eppendorf, Germany). The conditions for qRT-PCR were as follows. 3 min at 95 °C, 35 cycles of 95 °C - 3 s and 60 °C - 20 s in 96-well optical reaction plates (Axygen, USA). The amplicon specificity was verified by melt curve analysis from 60 to 95 °C after completion of 35 cycles. Each sample was replicated thrice. The comparative threshold cycle (C_T_) method was used to quantify the relative expression levels in real-time PCR. *OsActin1* was used as a reference gene to normalize gene expression. The ΔC_T_ for BPT-WT (control condition) was used as a control for the calculation of relative changes in expression in activation-tagged transgenic line (control), WT (NaCl stressed) and transgenic line (NaCl stressed). Relative expression level of genes was calculated using the ΔΔC_TT_ formula^81^. Primers used for gene expression studies and stress-responsive genes (*OsP5CS1, OsNAC6, OsLEA3, OsSOS1, OsZIP23, OsNHX1 and OsSalT*) are listed in Supplementary Table 1.

### Phylogenetic analysis of the *OsNF-YC13*

*Oryza sativa* NF-YC family protein sequences were obtained from Plant Transcription factor database (http://planttfdb.cbi.pku.edu.cn). Additionally, NF-YC family protein sequences of the wild rice species were obtained from NCBI/UniProt. Multiple sequence alignment was performed in ClustalW. The phylogenetic tree was constructed by Neighbor-joining method in MEGA 6.0 with default parameters of 1000 bootstraps.

### Isolation, cloning and subcellular localization of *OsNF-YC13*:GFP in onion epidermal cells

As *OsNF-YC13* has uninterrupted coding region, the coding sequence of *OsNF-YC13* was amplified directly from genomic DNA and fused in frame to the coding region of the N-terminus of GFP in the vector pRT35S-GFP vector to create the OsNF-YC13::GFP fusion protein under the control of the 35 S promoter. The OsNF-YC13::GFP construct was mobilized into *Agrobacterium* strain EHA105. We performed *Agrobacterium*-mediated onion epidermal peel transformation for studying the subcellular localization^82^. After 48 h co-cultivation, GFP localization was detected using a confocal laser scanning microscope (Carl Zeiss LSM880). The primers used for *OsNF-YC13* subcellular localization are given in Supplementary Table S1.

### Transactivation assay for NF-YC13 in yeast system

To assay the transactivation capacity of the NF-YC13 subunit, the coding region of OsNF-YC13 was amplified by PCR, subcloned into pGBKT7 (Clonetech, USA) and fused with the BD domain. The plasmid was confirmed by restriction enzyme analysis. The confirmed plasmid OsNF-YC13-pGBKT7 and pGBKT7 (control) were transformed into yeast strain PJ69–4A and plated on SD/-Trp medium for selection of positive colonies. Some colonies were picked and plated on SD/-Trp/-His and SD/-Trp/-Ade medium to check for the transactivation property of the subunit. At the same time, a serial dilution was performed by using liquid culture. For this, a colony was picked and inoculated in -Trp liquid medium. At 1.0 O.D, the cultures were serially diluted upto 10^−5^ and spotted on SD/-Trp, SD/-Trp/-His and SD/-Trp/-Ade plates for examining the transactivating property of OsNF-YC13 protein subunit. Empty vector was used as a control. Sequences of primers used are listed in Supplementary Table S1.

### *In-silico* analysis of upstream regulatory sequence of *OsNF-YC13*

To identify the presence of various *cis*-regulatory elements in *OsNF-YC13* gene, we extracted 1.5 kb upstream region from rice database RAP-DB (http://rapdb.dna.affrc.go.jp/) and analyzed using the online program PlantCare at bioinformatics.psb.ugent.be/webtools/plantcare^49^.

### Physiological parameters of activation-tagged line for salt stress treatment

#### Growth and weight of salt tolerant activation-tagged line

Growth parameters like shoot and root length and total dry weight were recorded after two weeks under control and NaCl stress treatment conditions. After recording the growth parameters, fresh weight of the seedlings was rapidly measured. Fresh samples were then dried in a hot air oven at 80 °C for 72 h and dry weights were measured separately.

#### Total chlorophyll content

Total chlorophyll content was estimated and calculated according to Lichtenthaler and Wellburn^83^. Twenty five mg samples of fresh leaves (150 mM NaCl treated) were incubated in 10 ml of 80% acetone and kept in dark for 48 h. The absorbance was measured at 663 and 647 nm for chl a and chl b respectively.

#### Estimation of proline content

The proline estimation was carried according to Bates *et al*.^84^. One gram of fresh leaf material was ground with 20 ml of 3% sulfosalicyclic acid (W/V) and the homogenate was centrifuged at 10,000 rpm for 10 min. The supernatant was used for the estimation of free proline. The reaction mixture comprised 2.0 ml of glacial acetic acid, 2.0 ml ninhydrin reagent and 2.0 ml of supernatant. The whole reaction mixture was boiled for one hour at 110 °C. After cooling the liquid to room temperature, 4 ml of toluene was added and mixed vigorously for 30 s on a cyclomixer. The chromophore (toluene) aspirated from the aqueous phase was taken and its absorbance was measured at 520 nm. Proline concentration in the samples was determined by a standard curve and expressed in terms of micro gram proline per one gram fresh weight.

### Estimation of Na^+^ and K^+^ ion content

For Na^+^ and K^+^ analyses, weighed dried samples of leaf and root (100 mg) were taken in digestion tubes containing 5 ml of tri-acid mixture, which consisted of nitric, perchloric and sulfuric acids (10:4:1). The tubes were subjected to 1 h digestion at 300 °C followed by overnight cooling. The digested liquid was then filtered through Whatmann No. 2 filter paper and the volume was made up to 50 ml with deioinized water. Sodium and potassium levels were analyzed using a flame photometer (ELICO-CL378, Type-223, India). The quantitative measurement of sodium and potassium ions was made using a standard curve.

### Extraction and estimation of antioxidant enzymes

Assay of antioxidant enzyme activities: The activity of antioxidant enzymes (superoxide dismutase (SOD), peroxidase (POX) and catalase (CAT)) were estimated in both leaf and root samples after two weeks of imposition of 150 mM NaCl stress.

### Enzyme extraction

The extraction procedure for SOD, POD and CAT was similar. Freshly weighed (100 mg) of leaf or root samples were frozen in liquid nitrogen to prevent proteolytic activity. The frozen tissues were ground using 5 ml of extraction buffer containing 100 mM phosphate buffer (pH 7.5) and 0.5 mM EDTA. The extract was centrifuged for 15 min at 12,000 rpm at 4 °C and the supernatant was used in the enzyme analyses.

### Superoxide dismutase activity (EC 1.15.1.1)

The SOD activity was measured according to Dhindsa *et al*.^85^. The reaction mixture comprised 3 ml each of methionine (200 mM), nitroblue tetrazolium chloride (NBT) (2.25 mM), EDTA (3.0 mM), riboflavin (60 μM), sodium carbonate (1.5 M), phosphate buffer (100 mM, pH 7.8) and the sample. The tube without enzyme was considered as control and the tube with enzyme considered as test. These tubes were kept under light (15 W) for 15 min followed by addition of enzyme and the absorbance measurement at 560 nm. The enzyme activity was expressed in [unit g^−1^(FW) min^−1^]. One unit of activity is the quantity of enzyme required to inhibit 50% initial reduction of NBT under light.

### Peroxidase (POD) activity (EC 1.11.1.7)

Peroxidase assay was carried out according to Castillo *et al*.^86^. The reaction mixture comprised 1.0 ml of phosphate buffer (pH 6.1), 0.5 ml guaicol, 0.5 ml H_2_O_2_, 0.1 ml of sample and the final volume was made up to 3 ml with double distilled water. Increase in the absorbance due to the formation of tetra-guaicol was recorded at 470 nm and it was expressed in μmol (guaicol reduced) g^−1^(FW) min^−1^.

### Catalase (CAT) activity (EC 1.11.1.6)

The catalase activity was measured according to Aebi^87^. The reaction mixture consisted of 1.5 ml phosphate buffer pH 7.0, 0.5 ml H_2_O_2_, 0.05 ml enzyme and the final volume was made upto 3 ml with distilled water. The Decrease in the absorbance was recorded at 240 nm and it was expressed in μmol H_2_O_2_ oxidized g^−1^(FW) min^−1^. All the observations on the absorbance were recorded in a double beam UV-VIS spectrophotometer (Spectrascan UV 2600, Chemito, India).

### Statistical analysis

All the recorded data were analyzed using Student’s *t*-test for statistical significance. **P < 0.01 and *P < 0.05 represent significant differences at 1 and 5% level respectively compared with the wild-type control.

## Electronic supplementary material

Supplementary information
