# Supplementary material for: Activation-tagging in indica rice identifies a novel transcription factor subunit, NF-YC13 associated with salt tolerance
Source: Sci Rep. 2017 Aug 24;7:9341. doi: 10.1038/s41598-017-10022-9 (PMC5570948; doi:10.1038/s41598-017-10022-9)
Supplement: Supplementary file 1 — Supplementary information [file 41598_2017_10022_MOESM1_ESM.doc]

**Supplementary Informations**

**Title:**

**Activation-tagging in *indica* rice identifies a novel transcription factor subunit, *NF-YC13* associated with salt tolerance**

Authors:

P. Manimaran^1#*^, S. Venkata Reddy^1#^, Mazahar Moin^1^, M. Raghurami Reddy^2^, Poli Yugandhar^2^, S.S. Mohanraj^1^, S.M.Balachnadran^2^, P.B. Kirti^1*^

**Supplementary Figures**

**Fig S1:**

Fig S1. (A) PCR analysis: Salt tolreant *Ds* lines were analyzed for presence of *Ac/Ds* or *Ds* elements by using *RFP:Nos* and *hpt* primers. Absence of *hpt* fragment indicates that the line carries the *Ds* element. M: 1 kb DNA ladder; B: blank: 1-18: *Ds* element carrying lines; C: WT-BPT; P: plasmid. (B): Representative picture of TAIL-PCR.

**Fig S2:**

Fig. S2: Distribution of genetically mapped insertions of *Ds* elements on corresponding chromosomes. The highest number of insertions was found on chromosome-1 (10). Red font indicate the corresponding *Ds* lines

**Fig S3:**

Fig. S3: Protein composition and Phylogenetic tree of OsNF-YC13 construction. A. Schematic protein structure of OsNF-YC13. B. Phylogenetic tree constructed using MEGA 6.0 software. Sixteen unique NF-YCs transcription factors from *O. sativa* along with NF-YCs from wild rice, *S. bicolor*, *S. italica, A.thaliana* and *G.max* were obtained from NCBI and PlantTFDB. The bar represents the changes in amino acid residue of the branch per unit length. The bootstrap values are represented at the braches. Note that the transcription factor NF-YC13 grouped to the transcription factors of wild rice. C. Amino acid alignment for OsNF-YC13 with its homologs in wild rice genotypes and other monocot. Alignment was performed by ClustalW. Asterisks indicate the identical aminoacids.

**Fig S4**

**A
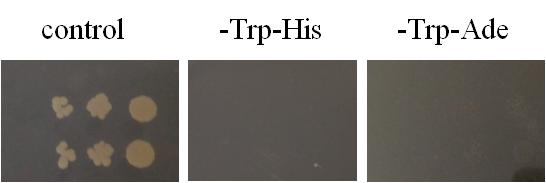
**

Transactivational activity of yeast cells expressing OsNF-YC13. OsNF-YC13 was fused to the GAL4 binding domain (BD, pGBKT7) and transformed into yeast cell PJ69-4A. Colonies were examined on –Trp-His and –Trp-Ade medium for transactivation assay. No transactivational activity was observed.

**B**
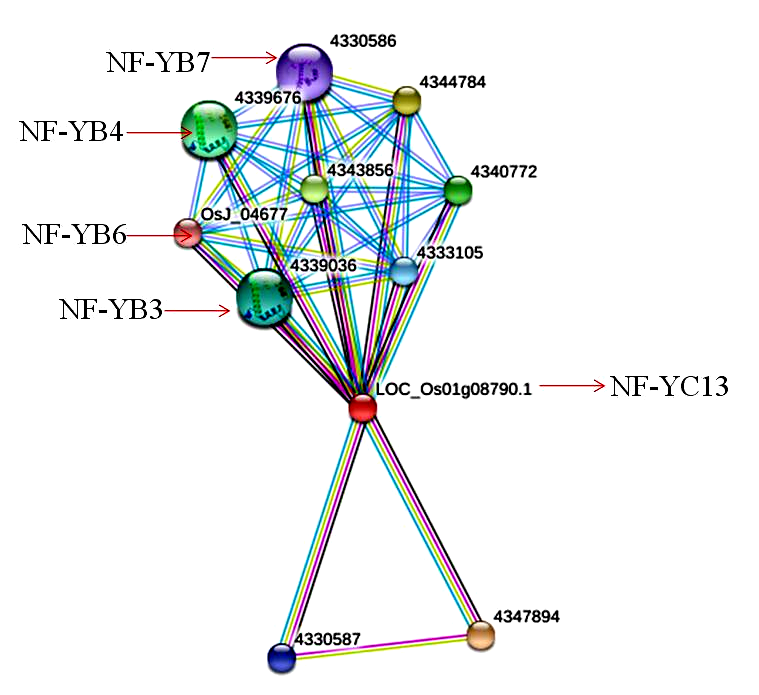


Identification of protein interaction networks with OsNF-YC13.

Using STRING online tool for identification of interaction protein partnes, the OsNF-YC13 might interact with NF-Y B subunits (B3, B4, B6, B7 and others).

**Supplementary Tables**

**Title:**

**Activation-tagging in *indica* rice identifies a novel transcription factor subunit, *NF-YC13* associated with salt tolerance**

Authors:

P. Manimaran^1#*^, S. Venkata Reddy^1#^, Mazahar Moin^1^, M. Raghurami Reddy^2^, Poli Yugandhar^2^, S.S. Mohanraj^1^, S.M.Balachnadran^2^, P.B. Kirti^1*^

**Supplementary Tables**

Table 1: Primers used in this study and their sequences (5′ to 3′)

| Gene Name | Forward primer | Reverse primer |
| --- | --- | --- |
| HPT | TATTTCTTTGCCCTCGGACGA | ATGAAAAAGCCTGAACTCACC |
| RFP-NOS | CCGACATCCCCGACTACAAG | CGCTATATTTTGTTTTCTATCGCGT |
| OsNF-YC13 (LOC_Os01g08790) | CGAGCCGATGGAGCAAG  (qRT-PCR) | TGGTCACCTTCTTGATGTCAC  (qRT-PCR) |
| OsNF-YC13  (For subcellular localization) | TATGGTACCATGGCCGCCGCCGCCGTC | CGCGGATCCCTAGGTGGTCTTCTGGAAGAACGC |
| OsNF-YC13 (Transactivation assay) | TATGAATTCATGGCCGCCGCCGCCGTCG (EcoRI) | CGCGGATCCCTAGGTGGTCTTCTGGAAGAACGC (BamHI) |
| Cytochrome p450  (LOC_Os01g08800) | CTCCTGCCATGGCTTCTC | GCTTGCTCCTCCTCCTAAAC |
| *OsP5CS1* | TGCCATCAGCACTAGAAAGG | ACAACCCATCCACATCACTG |
| *OsNHX1* | GCGGAAATCTCGCTCTCTTC | ACGAACAGGTTGATGGACAC |
| *OsSOS1* | AAGAGGACGAGGACAACTC | TTGGCTGGTCCAACAATTAC |
| *OsNAC6* | AGGACATCCTCATGTACTGG | TTCACCGGCCATGAAGC |
| *OsbZIP23* | TGATCCCTCGTTGCGTTAC | ACTCCAACCAACCAATCCC |
| *OsLEA3* | TCTTGGTGTGCAGGAGAAG | CGTCGCCTCCTTGGTATC |
| *OsSalT* | GGTGTGGATGGACAGGAATATG | GGGTTCCAGAAATCTCCTTGAT |
| *OsActin1* | CCGGTGGATCTTCATGCTTACCTGG | CGACGAGTCTTCTGGCGAAACTGC |

Table 2: Chromosomal localization, gene annotation of putative loci and flanking genes identified in salt tolerant *Ds* element carrying transgenic rice lines

| **Plant #** | **Chr #** | **region** | **Gene annotation** | **Protein** | **10 kb regions flanking the *Ds* element insertion** |
| --- | --- | --- | --- | --- | --- |
| DS-1 | 4 | Intragenic | LOC_Os04g52780 | leucine-rich repeat receptor protein kinase EXS precursor | Helix loop helix DNA binding protein, Gamma soluble NSF attachment |
| DS-2 | 3 | Intragenic | LOC_Os03g09910 | aminotransferase, classes I and II, domain containing protein | RNase L inhibitor protein, Sulphur transporter |
| DS-3 | 3 | Intragenic | LOC_Os03g28080 | RING finger protein | Transposon protein, expressed protein |
| DS-4 | 4 | Intragenic | LOC_Os04g51030 | wall-associated kinase 1 | Hypothetical protein, OsWAK50, OsWAK receptor like protein kinase |
| DS-5 | 1 | Intragenic | LOC_Os01g12660 | AAA-type ATPase family protein | Reticulon domain containing protein, expressed protein |
| DS-6 | 1 | Intragenic | LOC_Os01g04814 | vacuolar protein sorting-associating protein 4B | Expressed protein, vacuolar protein sorting-associating protein |
| DS-7 | 1 | Intragenic | LOC_Os01g03570 | transcription factor X1 | Multi copper oxidase type family protein, transposon protein |
| DS-8 | 4 | Intragenic | LOC_Os04g46700 | ABC family transporter: toluene tolerance | Thioesterase family protein, transposon protein unclassified |
| DS-9 | 1 | Intragenic | LOC_Os01g24710 | jacalin-like lectin domain containing protein | 60S ribosomal protein L23A, Expressed protein,Expressed protein |
| DS-14 | 1 | Intragenic | LOC_Os01g04650 | PB1 domain containing protein | Muconate cycloisomerase domain, reticulon domain containing protein |
| **DS-16** | **1** | **Intergenic** | **LOC_Os01g08800** | **Cytochrome P450** | **histone like transcription factor and archeal histone, cytochrome P450, expressed protein** |
| DS-18 | 1 | Intragenic | LOC_Os01g10940 | S-adenosylmethionine synthetase 2 | 4 copies of peptide N4-asparagine amidase A |
| DS-19 | 3 | Intragenic | LOC_Os03g61319 | RD26 (Similar to NAC transcription factor) | ATCHX, receptor like protein kinase, fumarylacetoacetase, hydrolase, alpha/beta fold family domain containing protein |
| DS-21 | 7 | Intragenic | LOC_Os07g40020 | GRAS family transcription factor domain containing protein | WD domain, G-beta repeat domain containing protein |
| DS-22 |  | Intragenic | LOC_Os01g72000 | armadillo repeat-containing protein | pyrroline-5-carboxylate reductase, rho-GTPase-activating protein-like, expressed protein |
| DS-25 | 1 | Intragenic | Loc_Os01g06230 | OsSAUR1 - Auxin-responsive SAUR gene family | gibberellin receptor GID1L2, gibberellin receptor GID1L2, retrotransposon protein, protein kinase, dirigent family protein (Disease resistance-responsive (dirigent-like protein), expressed protein |
| DS-27 | 2 | Intragenic | LOC_Os02g04490 | histone acetyltransferase HAC1 | cleavage and polyadenylation specificity factor, S-adenosyl-L-methionine-dependent methyltransferase mraW |
| DS-30 | 6 | Intragenic | LOC_Os06g48590 | CGMC includes CDA, MAPK, GSK3, and CLKC kinases | retrotransposon protein, macrophage migration inhibitory, CCT motif family protein factor |
| DS-33 | 1 | Intragenic | LOC_Os01g45750 | bile acid sodium symporter family protein | retrotransposon protein |
| DS-35 | 4 | Intragenic | LOC_Os04g40950 | glyceraldehyde-3-phosphate dehydrogenase | MYB family transcription factor, mitotic spindle checkpoint protein MAD2, OsFBX147 - F-box domain containing protein, DEAD-box ATP-dependent RNA helicase |

Table 3: Percentage identity between *OSNF*-YC13 and other monocot and dicot plants

| Name | % Identity |
| --- | --- |
| *OsNF-YC13* | ID |
| *O. rufipogon* | 100 |
| *O.nivara* | 99 |
| *S. bicolor* | 56 |
| *S. italica* | 55 |
| *G. max* | 17.4 |
| *A. thaliana* | 20.2 |

Table 4: Search of the promoter sequences of stress responsive genes up-regulated by OsNF-YC13 in AT-DS-16 line.

| Gene name | Locus name | Motif | Number of CCAAT element |
| --- | --- | --- | --- |
| *P5CS1* | Os05 g0455500 | CCAAT | 2 |
| *NAC6* | Os01g0884300 | CCAAT | 2 |
| *SOS1* | Os12g0641100 | CCAAT | 1 |
| *LEA3* | Os05 g0542500 | CCAAT | 1 |
| *SalT* | Os01g0348900 | CCAAT | 1 |
